# Mucin signature as a potential tool to predict susceptibility to COVID‐19

**DOI:** 10.14814/phy2.14701

**Published:** 2020-12-29

**Authors:** Mukulika Bose, Bhaskar Mitra, Pinku Mukherjee

**Affiliations:** ^1^ Department of Biological Sciences University of North Carolina at Charlotte Charlotte NC USA; ^2^ Idaho National Laboratory Idaho Falls ID USA

## Abstract

The Corona Virus Infectious Disease‐19 (COVID‐19) pandemic has played havoc on both the global health and economy. It is necessary to find a molecular signature that differentiates between low‐risk and high‐risk individuals. Pathogens, including viruses of the upper respiratory tract, utilize mucin proteins to enter into host cells. Mucins are critical components of innate immunity and also play important roles in infectious disease progression. Their expression is regulated by different cytokines during infection and inflammation. A comparison of mucin signatures between an asymptomatic versus symptomatic and between patients with mild versus severe symptoms could help identify other important proteins involved in the pathology of this new virus. Recent studies on the pathogenicity of the SARS‐CoV‐2 have found receptors that help its entry into the cells. In this review, we present an overview of how mucins are connected to the pathogenicity of the virus and propose that studying the glycome and mucin signature may lead to the development of a biomarker in predicting the susceptibility, progression, and response to therapy in COVID‐19 patients.

## Graphical abstract

The Corona Virus Infectious Disease‐19 (COVID‐19) pandemic has played havoc on both the global health and economy. A molecular signature to differentiate between low‐risk and high‐risk individuals will be helpful to set better quarantine measures. In this review, we present an overview of how mucins are connected to the pathogenicity of the virus and highlight the importance of studying the glycome and mucin signature in predicting the susceptibility, progression, and response to therapy in COVID‐19 patients.

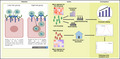

## 1 INTRODUCTION

The Corona Virus Infectious Disease‐19 (COVID‐19) pandemic has taken a toll on the world's population both in terms of health and economy. Since its first report in December 2019, in the city of Wuhan, China, it has infected more than 74 million people across the globe, with more than 1.6 million deaths worldwide (https://www.worldometers.info/coronavirus/country). In the United States alone, it has infected over 17 million people and killed more than 313,000 (https://www.worldometers.info/coronavirus/country/us/). Severe acute respiratory syndrome coronavirus (SARS‐CoV) is a respiratory pathogen that was first reported in the Guangdong Province of China in November 2002, that spread to 28 countries, infecting more than 8,000 people and had a mortality rate of 10% (Coleman and Frieman, 2013). The SARS‐CoV‐2 is another zoonotic virus that originated in bats and used an intermediary species (possibly pangolins) to switch hosts and infect humans (https://theconversation.com/study‐shows‐pangolins‐may‐have‐passed‐new‐coronavirus‐from‐bats‐to‐humans‐135687). The SARS‐CoV‐2 has spread to 218 countries now, with a mortality rate of about 3% worldwide. Different countries have taken mild to severe measures to abate the spread of the virus which include social distancing, stay‐at‐home orders, travel bans, etc., so that the health systems are not overwhelmed and consequently collapsed by the number of cases. The ideal is to take steps to flatten the pandemic curve so that the number of cases increases slowly and gradually thus lengthening the time to react. There is no solid evidence based on which the susceptibility, or disease progression can be predicted. The anti‐viral drug Remdesivir is under clinical trial (NCT04280705) and multiple mRNA vaccines are about to hit the market (Beigel et al., 2020; Jackson et al., 2020; Mulligan et al., 2020), but given the complexity and novelty of the infection, these may not be foolproof. Therefore, we should urgently find ways to identify groups of people who are the least and most likely to succumb to the disease and/or be symptomatic or asymptomatic carriers. The drastic impact of SARS‐CoV‐2 infection and the scarcity of dependable therapies have made it an emergency to explore the mechanisms of pathogenicity of the virus, starting from the viral entry into the human body to the subsequent pathophysiology and outcome.

Amidst the high propensity of research in finding an anti‐viral drug and vaccine, there is relatively less emphasis on finding ways to predict disease susceptibility, prognosis, and response to therapy. Population and epidemiologic studies clearly suggest that not everybody who gets infected with SARS‐CoV‐2 virus manifests the disease or needs to be hospitalized. Old age and chronic disorders seem to be the major risk factors for severe disease progression (Adams et al., 2020). A recent study has reported that the Reproduction Number (called R_0_) of this virus is between 4.7 and 6.6, which means that up to seven people can be infected from one infected person (Sanche et al., 2020). This number varies with viral load, immunity of the person infected, and several unknown factors. Thus, it is imperative to understand the plausible factors that define why some people remain asymptomatic, while others manifest with a range of symptoms from mild, moderate to severe.

## 2 SARS‐COV‐2 PATHOLOGY

SARS‐CoV‐2 is considered to be a phylogenetic sister to the SARS virus because the two share approximately 80% sequence similarity (Kong et al., 2020; Zhou et al., 2020; Zhu et al., 2020). The SARS virus was aggressive, and patients mostly showed symptoms within 2–3 days. However, SARS‐CoV‐2 spreads faster and about 50% of the patients do not show symptoms, thus spreading the virus without their knowledge. Also, for people who do show symptoms, it takes 7–14 days to manifest, thus giving the virus ample time to be transferred from person to person. The advent of summer weather and severe quarantine measures might have resulted in the disappearance of the SARS virus in 2003, but these do not seem to apply to the new SARS‐CoV‐2.

In order to enter into cells, coronaviruses bind to a cell surface receptor for attachment, subsequently entering the endosomes, and then fusion of the viral and lysosomal membranes occur. Similar to the SARS‐CoV, the entry of SARS‐CoV‐2 into cells occurs by the binding of the receptor‐binding domain (RBD) of the viral spike (S) glycoprotein, that is a part of the viral envelope, (Hoffmann et al., 2020; Walls et al., 2020) to the angiotensin‐converting enzyme 2 (ACE2) receptors present on various human tissues, including but not limited to the heart, blood vessels, gut (intestinal epithelial cells), lung, kidney, testis, and brain (Kuba et al., 2013; Verdecchia et al., 2020). The RBD keeps switching between two positions, standing‐up for receptor binding and lying‐down to evade immune response (Gui et al., 2017; Yuan et al., 2017). Subtle functionally important differences between the crystal structure of SARS‐CoV and SARS‐CoV‐2 enable the SARS‐CoV‐2 RBD to have a significantly higher binding affinity to ACE2 (Shang et al., 2020a). However, the SARS‐CoV‐2 RBD, albeit more potent, is found to be mostly present in the lying down state, thus being less exposed than the RBD of SARS‐CoV (Walls et al., 2020; Wrapp et al., 2020). The entry of SARS‐CoV‐2 also depends on the activity of TMPRSS2 protease found on alveolar epithelial cells that activates the S protein (Hoffmann et al., 2020), and preactivated by proprotein convertase furin, thus reducing its dependence on proteases of the host cells for entry (Shang et al., 2020b). These molecular characteristics help the SARS‐CoV‐2 to efficiently enter the cells, while evading the immune system (Shang et al., 2020b). All these factors make SARS‐CoV‐2 a more dangerous pathogen.

SARS‐CoV‐2 enters through the nasopharyngeal epithelial cells and progresses into the lungs. The immune cells secrete cytokines to keep the immune system stimulated, in turn, leading to excess secretion of mucus. This causes severe inflammation, and lack of oxygen circulation in the lungs thus clogging the alveoli leading to shortness of breath. Recent evidences have suggested that it may cause other lethal problems like heart attack, acute kidney disease, brain damage, blood clots, intestinal damage, and liver problems. Some patients start accumulating a lot of inflammatory fluids in lungs, a condition called acute respiratory distress syndrome (Ruan et al., 2020). In this condition, oxygen levels are reduced creating shortness of breath for the patient. A phenomenon called “cytokine storm” takes place in which the immune cells produce too many cytokines to further activate the immune system (Mehta et al., 2020). Cytokines can also damage normal cells, causing leakage of blood vessels, blood clots, and sharp decrease in blood pressure (https://www.welthi.com/beyond‐lungs,‐covid‐19‐can‐cause‐damage‐throughout‐the‐body/).

## 3 ROLE OF MUCIN GLYCOSYLATION IN INFECTION AND IMMUNITY

In order to survive the external environment, most mammals, including humans use complex molecules that make up a thick layer of mucus and act as a protective barrier. This epithelial barrier is made up of the mucin proteins that act as the first line of defense and is part of our innate immunity, in which glycans play a critical role in cell–cell adhesion and communication (Zhao et al., 2008). Mucins are mainly produced by surface goblet cells and glandular epithelial cells, that are connected to other parts of the innate and adaptive immune systems. Mucins are heavy transmembrane and secreted heterodimeric glycoproteins, and their degree of glycosylation determines their protective function (Bose & Mukherjee, 2020). Mucins are present on almost all epithelial cells lining the respiratory, gastrointestinal, and reproductive organs. They are mainly made up of O‐glycosylated repeats which bind water and give them their characteristic gel‐like properties (Corfield, 2015). Some mucins are membrane‐bound having a hydrophobic membrane‐spanning domain that favors adherence to the plasma membrane. Most mucins are secreted as gel‐forming mucus or to form a component of saliva. Mucins are composed of an extracellular N‐terminal domain that is glycosylated and participates in ligand binding and cell‐cell adhesion, and an intracellular C‐terminal domain that has highly conserved phosphorylation sites and binds to various proteins and transcription factors thus playing major roles in downstream signaling (Martín et al., 2019). They undergo biochemical changes both in the extracellular domain and the cytoplasmic domain during bacterial, viral and parasitic infections and directly influence pro‐inflammatory and anti‐inflammatory responses (Bose & Mukherjee, 2020; Dhar & McAuley, 2019). Mucins sense ligands of pathogenic origin and pass this information downstream by activating immunomodulatory pathways. Currently, 22 human mucin genes have been identified which are divided into two major types, membrane‐bound and secretory. In humans, membrane‐bound mucins include MUC1, MUC3A, MUC3B, MUC4, MUC12, MUC13, MUC16, MUC17, and MUC22, and secreted mucins include MUC2, MUC5B, MUC5AC, MUC6, MUC7, MUC19, and MUC20 (Martín et al., 2019). Human mucin genes MUC21 and MUC22 have been recently identified and are located near to each other (Norman et al., 2017).

Mice lacking both copies of mucins like MUC1, MUC2, MUC3, and MUC16 have been shown to have increased bacterial infection and inflammation (Linden et al., 2008). Previously, it has been shown that MUC1 double knockout mice infected with Influenza‐A virus were more prone to infection compared to wild‐type mice (McAuley et al., 2017). This study shows that MUC1 is a critical component of the host immune response that keeps the severity of influenza in check. An increase in MUC15 expression was found to coincide with the peak of immune activation during influenza (Chen et al., 2019a). Theories of glycobiology state that changes in host glycosylation can cause inflammation, and inflammatory signaling induced by infection may lead to changes in host glycosylation. This is described as the “glyco‐evasion hypothesis” (Kreisman & Cobb, 2012). These studies provide the rationale for studying mucins to elucidate mechanisms of infectious disease pathologies.

Many knockout mouse models with glycosylation changes have been generated over the decades. In mice, some of these knockouts are embryonic or newborn lethal (Marek et al., 1999; Shafi et al., 2000; Wang et al., 2001; Ye & Marth, 2004), which is also seen in the human population by the low survival rate and severe pathologies associated with the congenital disorder of glycosylation (CDG) diseases (Freeze & Ng, 2011; Jaeken, 2010). These data show the physiological relevance of glycans in general and that the disturbance of glycosylation enzymes generates immune‐associated pathologies. Sialylated core 1 O‐glycans are considered a hallmark of naïve CD8^+^ T cells present in the thymus; however, the loss of the sialylated core 1 and the synthesis of core 2 O‐glycans are found during the activation of CD8^+^ T cells in the periphery (Chervenak & Cohen, 1982; Piller et al., 1988). The O‐glycans revert to the core 1‐dominated phenotype after the differentiation into CD8^+^ memory cells, (Galvan et al., 1998) and those effector CD8^+^ T cells still having core 2 O‐glycans undergo apoptosis. Animals without the ST3 Gal‐I sialyltransferase enzyme were reported to lack the sialylated core 1 O‐glycans on naïve CD8^+^ T cells (Priatel et al., 2000). Also, the loss of ST3 Gal‐I enhanced synthesis of the core 2 O‐glycans on naïve T cells in the periphery, thus leading to apoptosis in the absence of immune challenge (Priatel et al., 2000). These results show that glycoprotein sialylation is a homeostatic mechanism for CD8^+^ T cell‐dependent immune responses. The removal of the ST6 Gal‐I enzyme in mice resulted in a severe B cell‐centered phenotype where IgM levels and proliferation of B cells were significantly reduced upon stimulation via IgM or CD40 cross‐linking. Antibody responses were also reduced for both T‐independent and T‐dependent antigens (Hennet et al., 1998).

The evidence of bacterial infection‐mediated glycosylation changes are many and have been reviewed previously (Kreisman & Cobb, 2012). However, even viruses are known to interact with glycans to enter into host cells (Bose & Mukherjee, 2020). Hemagglutinin is a viral protein that binds to glycans containing sialic acids on the target cell surface and aids viral adherence to the cell. The viral‐encoded enzyme neuraminidase cleaves these host sialic acid molecules upon entry, leading to the detachment of viral particles into the cell. This is a crucial event for the viral lifecycle, since neuraminidase inhibition can be an effective therapy (Grienke et al., 2012). In this context, it becomes imperative that glycosylation patterns of mucins influence susceptibility to infection, magnitude of immune response, and to some extent, response to therapy.

## 4 CONNECTION OF MUCINS TO COVID‐19

With regard to the deployment of immune cells and the production of immunoglobulins, the mucosal immune system is the largest component of the innate immune system. The mucosal immunity has evolved to provide protection at the sites of infection providing a mucous membrane and secretory IgAs are mucosal antibodies that are produced in higher quantities than all other isotypes combined (Russell et al., 2015a, b).

Many studies have analyzed the vital role that mucins play in infectious diseases including COVID‐19 (Bose & Mukherjee, 2020; Plante et al., 2020). We already know that aged, immunocompromised, and people with chronic disorders are the worst affected. This prompted us to provide our perspective on how studying the mucin signature can be helpful in predicting the susceptibility, progression, and response to therapy in COVID‐19 patients and segregate them into high‐risk and low‐risk groups.

Other than binding to their specific receptors, viruses utilize the transmembrane glycoproteins (including mucins) to enter into epithelial cells. Therefore, glycosylation profiles of the mucins in patients will be a useful tool in finding a pattern to predict infection susceptibility and disease progression.

**FIGURE 1:**
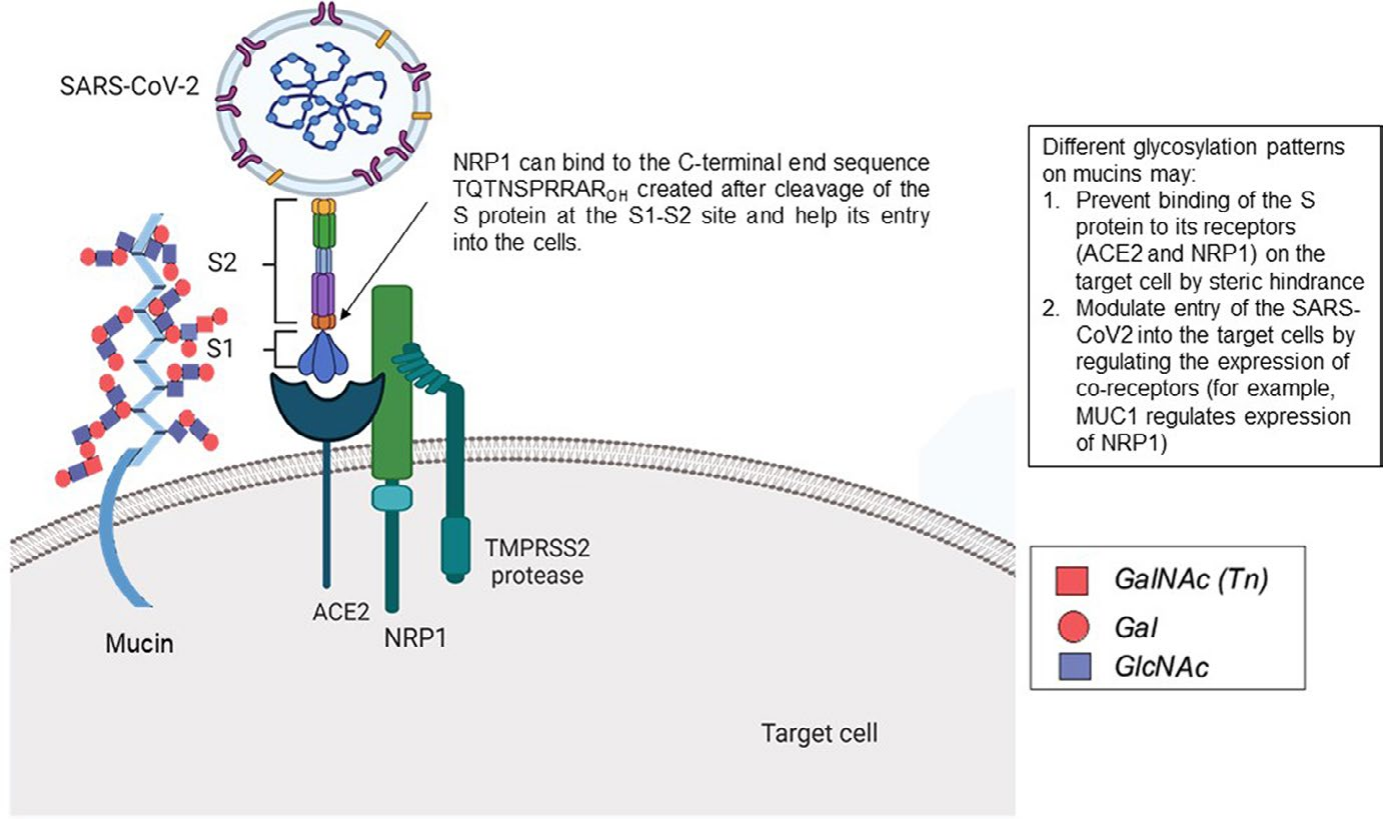
Schematic diagram showing the role of mucins in susceptibility toward COVID‐19. Different glycosylation patterns on mucins may aid or prevent the entry of SARS‐CoV2 into the target cells. Different glycosylation patterns on mucins may (1) prevent the binding of the S protein to its receptors (ACE2 and NRP1) on the target cell by steric hindrance and (2) modulate entry of the SARS‐CoV2 into the target cells by regulating the expression of co‐receptors (for example, MUC1 regulates expression of NRP1). The diagram was created with Biorender and Autocad

The outer layer of the airway epithelial cells contains gel‐forming mucins (MUC5AC and MUC5B), while the inner layer consists of membrane‐tethered mucins (MUC1, MUC4, and MUC16) that are occasionally shed from the apical cell surface (Kim, 2012). During the infection of the airways, these mucins act as a protective barrier against pathogens. Mucins also serve as a binding site for various pathogens (Bose & Mukherjee, 2020), and might help entry and/or exit of SARS‐CoV‐2. Differential and specific glycosylation patterns on certain mucins may restrict/enhance the binding of virus to its respective receptor on epithelial cells by various mechanisms including steric hindrance, as depicted in Figure 1. We, therefore, hypothesize that the glycome signature and signature of shed mucins in circulation from infected lung or respiratory tract epithelial cells may correlate with the outcome of viral infection and disease progression.

MUC4 protein has been demonstrated to protect female but not male mice from SARS‐CoV‐2 (Plante et al., 2020). The study identified nine quantitative trait loci (QTLs) impacting SARS‐CoV disease by screening the genetically diverse collaborative cross (CC) mouse population founders and cell lines (Churchill et al., 2004). Among these QTLs, HrS2 was located on chromosome 16, which was found to modulate SARS‐CoV titer levels in the lung (Gralinski et al., 2015). The HrS2 locus was narrowed to a priority candidate gene, MUC4, by the integration of sequence data from the CC founders and gene expression results from their preCC experiment (Keane et al., 2011). In mice containing the PWK/PhJ allele, low expression of MUC4 corresponded with low SARS‐CoV titer associated with the QTL at locus HrS2. On the examination of infection in mice lacking MUC4, it was found that MUC4 does not regulate SARS‐CoV titer. However, it plays a role in attenuating the pathogenicity of viral infections (Plante et al., 2020). Females lacking MUC4 showed consistently increased susceptibility to SARS‐CoV‐induced disease, but males did not. It has been reported that SARS‐CoV causes more severe disease in male mice and that the females partly derive their resistance from estrogen signaling (Channappanavar et al., 2017). Likewise, epidemiological data from both the SARS viruses suggest that human females may be more resistant than human males (Chen et al., 2020; Huang et al., 2020; Karlberg et al., 2004; Leong et al., 2006). Estrogen enhances MUC4 transcription in a tissue‐specific manner (Lange et al., 2003), this may be a reason why the absence of MUC4 has greater impact on female mice. Males have been shown to be worse affected than females and a theory is that estrogen in females plays a role in dampening the cytokine storm and shedding of epithelial mucins which clog the alveoli and cause shortness of breath. Further, MUC1 stabilizes estrogen receptors (ER) and is immunosuppressive. The association between MUC1 C‐terminal domain and ERα was found to be stimulated by 17β‐estradiol (E2), in breast cancer cells. MUC1 binds directly to the DNA binding domain of ERα and stabilizes it by inhibiting its ubiquitination and subsequent degradation (Wei et al., 2006). A recent study reported markedly increased levels of MUC1 and MUC5AC in the sputum aspirated from the trachea of patients with severe COVID‐19 symptoms (Lu et al., 2020). All the 16 patients recruited in this study were admitted to the intensive care unit because of low oxygenation index, and most of them received mechanical ventilation. Blood tests revealed an elevated inflammatory index in most of these patients, including leukocyte count, C‐reactive protein, and interleukin‐6. Optical coherence tomography showed clear bronchiole in healthy controls and mucus retention in the bronchiole of COVID‐19 patients. High viscosity white to gray sputum were aspirated from the respiratory tracts of these patients, whereas, the induced sputum from healthy controls were clear and transparent with low viscosity. Airway mucus from COVID‐19 patients had a higher level of MUC5AC, MUC1, and MUC1‐CT fragment compared to that of healthy controls. More than half of these patients presented with a dry cough, however, this study provided direct evidence of mucus retention in their small airway, and they were incapable of expectorating by themselves and needed bronchoscopy aspiration to clean the respiratory tract. High levels of the gel‐forming MUC4 dehydrates airway surfaces and leads to mucus adhesion, thus causing shortness of breath. All the 16 critically ill patients recovered owing to an aggressive clearance of the respiratory tract by the medical practitioners (Lu et al., 2020). Therefore, it is plausible that high levels of shed mucins correspond to worse outcome of the disease and this must be investigated. It is also possible that certain treatments be more potent in patients with expression of specific mucins and their glyco‐pattern.

Another study reported increased expression of innate immunity genes and carbohydrate metabolism genes in goblet cells which are also known as mucin secretors (Sungnak et al., 2020). Multiple scRNA‐seq datasets were generated within the Human Cell Atlas consortium (HCA), and it was found that SARS‐CoV‐2 entry receptor ACE2 is expressed more, and also co‐expressed with viral entry‐associated protease TMPRSS2, in nasal epithelial cells, and more specifically in goblet and ciliated cells. This study indicates that these cells are possible reservoirs for dissemination within a patient and from person to person. Also, viral infection itself could drastically alter the gene expression profile in the nose and other tissues. In highly exposed nasal epithelial cells, there was an up‐regulation of innate immune genes in association with ACE2. Association of carbohydrate metabolism genes with ACE2 expression was also found, probably because goblet cells make mucins. Therefore, it would be interesting to investigate how glycosylation patterns might affect the susceptibility of these cells to infection due to their association with viral receptor expression. Since SARS‐CoV‐2 is an enveloped virus, it does not require cell lysis for release. Therefore, the virus might exploit secretory pathways in nasal goblet cells for slow, continuous‐release in the early stages with no symptoms (Sungnak et al., 2020).

Recently, an article published in “*Science*” has reported that neuropilin‐1 (NRP1), known to bind furin‐cleaved substrates, significantly potentiates SARS‐CoV‐2 infectivity and a monoclonal antibody against NRP1 blocked this effect (Cantuti‐Castelvetri et al., 2020). A mutant SARS‐CoV‐2 with an alteration in the furin cleavage site did not depend on NRP1 for infectivity. Human COVID‐19 autopsies were subjected to pathological analysis, which revealed that NRP1 was expressed on SARS‐CoV‐2 infected cells including olfactory neuronal cells facing the nasal cavity (Cantuti‐Castelvetri et al., 2020). Interestingly, MUC1 has been reported to increase the levels of neuropilin‐1 (NRP1), a co‐receptor of vascular endothelial growth factor receptor (VEGFR) and its ligand, VEGF (Wild et al., 2012; Zhou et al., 2016). Blocking the interaction between VEGF_165_ and NRP1 with an anti‐NRP1 heptapeptide A7R (ATWLPPR), significantly reduced VEGFR signaling and tumor growth in a pancreatic ductal adenocarcinoma mouse model (Zhou et al., 2016). Therefore, MUC1 might play an important role in the pathology of SARS‐CoV‐2 (Figure 1) and A7R could be a potential drug to prevent viral entry into the cells. However, blocking Neuropilin directly might cause side effects, hence proper investigation needs to be performed to inhibit the specific interaction between the virus and NRP1.

As in the case of previous pandemics of SARS and MERS (Middle Eastern respiratory syndrome), corticosteroids are not recommended on a daily basis and might worsen COVID‐19‐associated lung injury (Russell et al., 2020). However, in case of hyperinflammation, immunosuppression is a possible strategy. A phase 3 randomized controlled trial of IL‐1 blockade (anakinra) in sepsis, was found to be significantly associated with a survival benefit in patients with hyperinflammation, without adverse effects (Shakoory et al., 2016). In China, another randomized controlled trial of tocilizumab (IL‐6 receptor blocker for cytokine release syndrome), has been approved in COVID‐19 patients with pneumonia and elevated IL‐6 (ChiCTR2000029765) (Fu et al., 2020; Wu et al., 2020). There are many studies reporting the feedback loops between cytokines like IL‐1β, IL‐6, etc., and the production of mucins during infections and inflammations (Chen et al., 2019b; Neveu et al., 2009; Sharba et al., 2019). Inhibition of Janus Kinase (JAK) could affect both viral entry into the cell and inflammation (Richardson et al., 2020). The involvement of the JAK‐STAT pathway in the regulation of mucin production has been reviewed previously (Theodoropoulos & Carraway, 2007). Therefore, it is apparent that there is a huge yet unelucidated role of mucins in the pathophysiology of SARS‐CoV‐2. And studying the mucin signature with respect to COVID‐19 pathophysiology will also help surface many other receptors and proteins as potential targets to combat the disease. This may also lead to the development of mucosal vaccines to prevent infection (Russell et al., 2020).

## 5 CONCLUDING REMARKS AND FUTURE IMPLICATIONS

**FIGURE 2:**
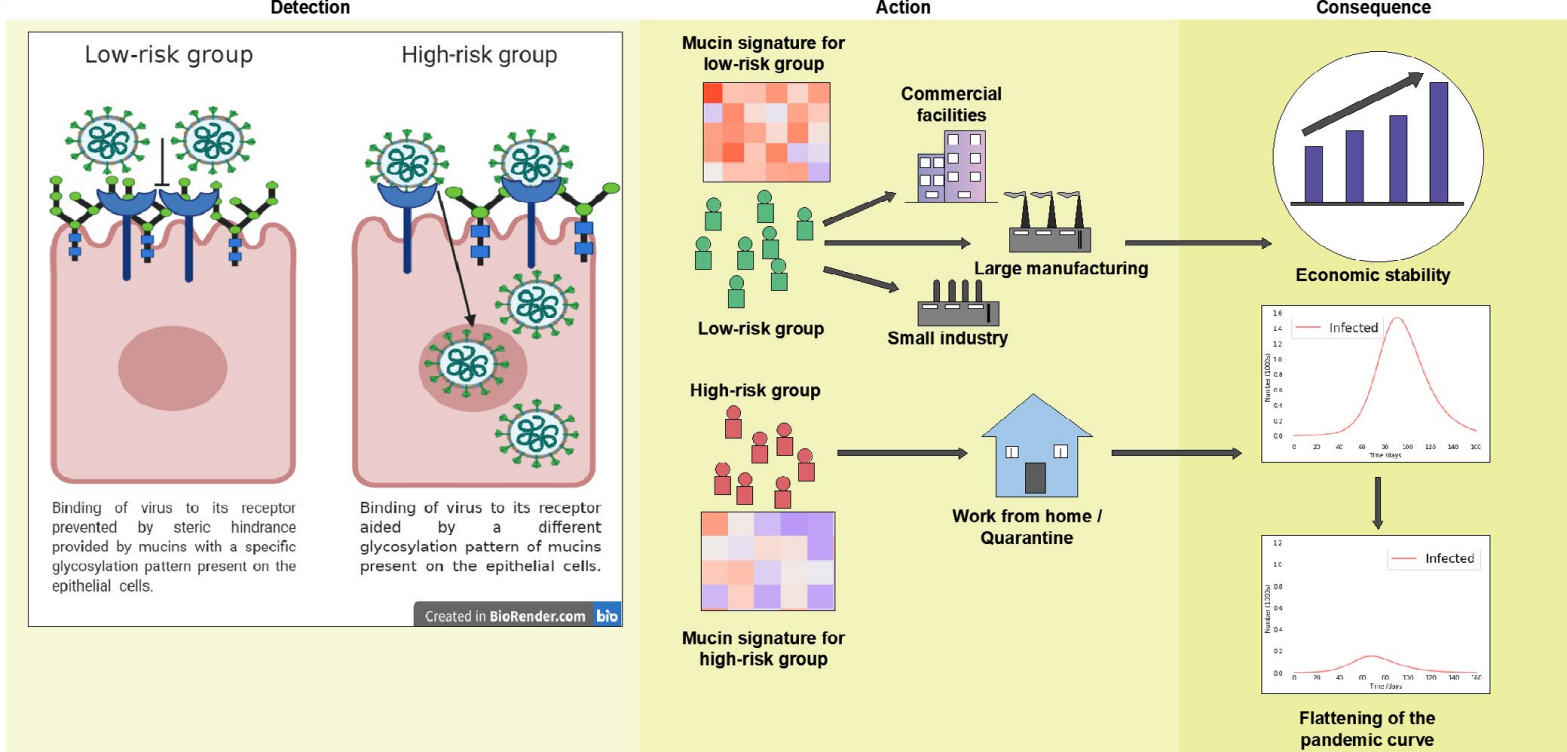
Schematic diagram showing the role of mucins in susceptibility toward COVID‐19. Detection: On the left‐hand side, an epithelial cell of a low‐risk individual shows how the binding of SARS‐CoV2 to the ACE‐2 receptor (shown in blue) is prevented due to steric hindrance by the mucins (shown in green). On the right‐hand side, an epithelial cell of a high‐risk individual shows how the different glycosylation of the mucins (shown in green) aid in binding of the SARS‐CoV2 to ACE‐2 and entry into the cells. Action: Glycome signature of mucins should be used to distinguish between high‐risk and low‐risk groups and will enable the implementation of better quarantine rules in the coming days. Consequence: This will not hamper work and therefore the economy will be stable and also high‐risk groups working from home will help flatten the pandemic curve

In conclusion, there is no standard treatment for COVID‐19 and the variability of symptoms, and their differential manifestation pose great risks to the vulnerable. Although multiple mRNA vaccines will soon be available, the best way to prevent the collapse of healthcare facilities is to identify high‐risk versus low‐risk groups. The aim of this article is to provide a rationale for conducting research on the role of mucins in the pathophysiology of COVID‐19 patients. This holds great potential to establish a glycome signature as a biomarker to help predict disease susceptibility, disease progression, and response to therapy (Figure 2).

The levels of shed mucins from normal and SARS‐CoV‐2 infected lung epithelial cells should be tested to find the viral load necessary to establish infection and epithelial cell damage. Blood and serum should be tested from patients with both symptomatic (mild, moderate, severe) and asymptomatic disease for the amount and type of shed mucins. This will establish the shed mucin signature for symptomatic versus asymptomatic carriers. Saliva and nasopharyngeal fluids from these patients should be studied for the glycome pattern (the collection of all glycoproteins in a cell) to establish the glycome signature which distinguishes a “super‐spreader” from other patients or a symptomatic from an asymptomatic carrier (Sapkota et al., 2020). The glycome signature of uninfected, healthy volunteers needs to be included to predict susceptibility. Further, we hypothesize that the pattern of mucin glycosylation on the epithelial cells may help understand the differential internalization of the virus. Since it is known that the spike protein of SARS‐CoV‐2 binds to ACE2 receptors to enter cells with the help of the protease TMPRSS2, the correlation and/or causal relationship between the major mucin glycopattern and ACE2 and TMPRSS2 should be established. Mucin glycopattern from bronchoalveolar lavage fluid (BALF) from each group can be easily correlated to the pro/anti‐inflammatory cytokines by proteomic analysis to determine the magnitude of inflammation (cytokine storm), the hallmark of COVID‐19 deaths. To predict the severity of disease in humans, the saliva and nasal mucus can be collected, and the glycosylation pattern detected. A comparative analysis between the mucin glycosylation profiles of patients with mild versus severe symptoms will generate a signature to predict the prognosis of disease. Also, the same methods can be used to generate a signature for the prediction of outcome to therapy. Studying the glycosylation profile of mucins from the saliva and nasal mucus from the patients will be clinically more relevant compared to using mouse models. Long‐term research to directly implicate which mucin subtype/s play key roles in the severity of the disease, relevant immunocompetent mouse models with specific mucin genes or multiple mucin genes knocked out can be employed and disease susceptibility and progression to acute lung damage studied.

Among several diverse challenges with this pandemic, two primary ones that surface to the top are (1) lack of an underlying cellular mechanism that delineates asymptomatic patients versus patients with mild, moderate, and severe symptoms and (2) varying response of patients to a standard treatment regimen.

Recently, an article has urged more research on mucosal immunity in COVID‐19 and how this may lead to the development of a mucosal vaccine (Russell et al., 2020). Understanding how the mucin signature distinguishes an asymptomatic spreader from a symptomatic spreader or a highly susceptible individual to one with low susceptibility will be helpful to determine the population that is at high‐risk. Studying the mucosal immunology axis will also reveal more information on infection‐induced cancers and might provide insight into how infection with COVID‐19 may affect other chronic inflammatory diseases like cystic fibrosis, asthma, cancer, etc. This type of analysis will consider the disparity that we see in different human races and can also be extended to study susceptibility in other animal species, for example, dogs that are being suspected to have contracted the disease from their owners. The identification will aid in making better policies for new work‐related quarantine measures for the vulnerable people, thus saving time, money, and effort in combating the COVID‐19 pandemic and other future global outbreaks.

## CONFLICT OF INTERESTS

The authors declare no conflicts of interest.

## AUTHORS’ CONTRIBUTIONS

MB and PM conceived the idea and prepared the manuscript; BM created the figures.
